# Predictors of adherence to option B+ approach for the prevention of mother to child transmission of human immunodeficiency virus in Abuja, 2017

**DOI:** 10.11604/pamj.2021.38.54.16690

**Published:** 2021-01-18

**Authors:** Augustine Olajide Dada, Aisha Abubakar, Adebobola Bashorun, Patrick Nguku, Abisola Oladimeji

**Affiliations:** 1 Nigeria Field Epidemiology and Laboratory Training Program Abuja, Abuja, Nigeria,; 2 Ahmadu Bello University Zaria, Zaria, Nigeria

**Keywords:** HIV/AIDS, adherence, antiretroviral therapy, Nigeria

## Abstract

**Introduction:**

option B+ ART is a lifelong regimen of ART using a combination of 3 ARVs and adherence to this regimen can reduce risk of MTCT to 1-2% as against 15-40% without treatment. To achieve an undetectable viral load and prevent the development of drug resistance, a person on ARV drugs need to take at least 95% of prescribed doses on time. This study assessed the level of adherence to Option B+ PMTCT program and its predictors among HIV+ Pregnant women accessing antenatal care in health facilities Abuja.

**Methods:**

we enrolled 284 HIV positive pregnant women and lactating mothers in a hospital-based cross-sectional study. We sampled respondents using two-staged sampling technique. We collected data on socio-demographic characteristics, level of adherence, patients and healthcare related factors affecting adherence, knowledge of clients on HIV, ART and MTCT. Focused group discussion guide, data abstraction form and key informant interview guide were used for PMTCT focal persons. We conducted bivariate analysis and logistic regression using Epi-Info version 7 at 5% level of significance.

**Results:**

the mean age of respondents was 30.12 years (SD±4.86) with mean knowledge score of 16.7 and 75.5%% of them had good knowledge. The level of good adherence was 83.3%. Independent factors associated with non-adherence to ART included: Forgetfulness (OR 20.02; 95% CI 6.42-62.48), having side effects (OR 39.6; 95% C.I: 4.46-352.32), lack of food (OR 34.76; 95% C.I: 2.37-509.33), disclosure of HIV status (OR 2.51; 95% CI 1.22-5.15), being too busy (OR 13.96; 95% CI 3.89-49.98). Encountering challenge in ART initiation (OR 2.05; 95% CI 1.01-4.72) and level of Knowledge (OR 2.12; 95% CI 1.06-5.42).

**Conclusion:**

the level of adherence would improve study if the Public health department of Federal Ministry of Health (FMOH), Federal Capital Development Authority (FCDA) and National Agency for the Control of AIDS (NACA) sponsors public enlightenment on HIV/AIDS through the media which may help reduce stigma and encourage voluntary HIV status disclosure. Reminders should be used by patients to help them overcome forgetfulness.

## Introduction

HIV/AIDS poses a major public health and developmental challenge in the world, more so in sub-Saharan Africa [1]. Globally, most new infections occur in Africa with mother to child transmission accounting for 90%, and a risk of 15-40% without interventions [2]. The World Health Organization (WHO) has implemented different strategies for the optimization of the prevention of the mother to child transmission (PMTCT) care and support: Option A, Option B, and Option B+ [3]. Option B+ antiretroviral therapy is a life-long regimen of ART for pregnant and breastfeeding women, commencing at the time of HIV diagnosis regardless of CD4 count or clinical stages using a combination of three antiretroviral drugs (ARVs). Adherence to this regimen can reduce risk of Mother-to-Child-Transmission to 1-2% [3,4]. Treatment adherence is defined as the extent to which a person currently takes prescribed medications [4]. The effectiveness of antiretroviral drugs in viral load suppression and reducing the Mother-to-Child-Transmission of HIV is highly dependent on adherence [5]. A WHO report has shown that in women with higher adherence levels to ART, over 80% of their children were protected from HIV, as opposed to a lower percentage among women with a lower adherence rate. To achieve an undetectable viral load and prevent the development of drug resistance, a person on ARV drugs need to take at least 95% of prescribed doses on time. There is no gold standard to measure adherence, commonly used method includes patient self-report and pill count.

Poor adherence increases the potentials of virologic failure, maternal HIV disease progression, and risk for development of drug resistance, which all lead to an increased risk of MTCT. Poor adherence is mostly responsible for poor treatment outcomes among people receiving ART. Apart from directly affecting mothers´ well-being, poor adherence may hasten the development of drug resistance thereby resulting in a shift from first-line regimens to more expensive second-line regimens at an early stage. One of the main goals of the global plan is to reduce the rate of mother to child HIV transmission to less than 5%. Nigeria is however facing various challenges regarding adherence to ART, as high percentage of pregnant mothers do not adhere to the use of lifelong treatment for different reasons. This study was therefore conducted to assess the level of adherence to ART and factors influencing adherence among HIV positive pregnant and breastfeeding women receiving care in selected health facilities within the Federal Capital Territory (Abuja). Also, providing insight into adherence to the option B+ treatment approach; a recent programme commenced in Nigeria, in September 2015.

## Methods

We conducted a cross sectional survey among HIV positive pregnant and breastfeeding women, enrolled on the option B+ approach of PMTCT care and treatment in selected healthcare facilities in Abuja. Abuja is the capital city of Nigeria, with a projected total population of 3 166 506 (2016 population projected from 2006 census). This study was carried out in seven General Hospitals (General Hospital Kubwa, General Hospital Bwari, General Hospital Karshi, General Hospital Gwarimpa, Wuse District Hospital, Maitama District Hospital) providing comprehensive HIV services which include HIV testing services (HTS), prevention of mother to child transmission (PMTCT), antiretroviral therapy (ART) for adult and children, support group services and treatment of opportunistic infections. We calculated the sample size for the study using a prevalence of adherence to antiretroviral therapy from a previous study [6], the sample size was approximated to 284. A two-staged sampling technique was used to select the participants for the study. Seven health facilities were selected by simple random sampling and proportionate allocation of respondents was done based on the number of women registered on PMTCT services in the selected health facilities. Respondents were selected using the number of clinic attendees in the register as the sampling frame, an interval or fraction was generated which was used in the selection. We administered the questionnaire over ten weeks as PMTCT clinic runs twice in a week (20 clinics within 10 weeks), sampling two to three respondents per clinic day. Clinic attendees in the register were assigned numbers from 1-N, then a sampling interval was determined by dividing the study population and size of sample N, that is, 6/2= 3. The first sample was chosen randomly by balloting between 1 and 3, and then subsequently every 3rd patient was selected. The purpose of the study was explained to the identified participant and the study questionnaire was administered to those who consented.

Eligible participants were HIV positive pregnant and breastfeeding women aged 15-49 years, who were enrolled into the hospital HIV/AIDS care program, having been on antiretroviral drugs for at least 3 months to commencement of data collection and attending the antenatal/postnatal ART clinics. The dependent/outcome variable of interest was adherence to option B+ PMTCT drugs, while the independent/exposure variables were socio-demographic characteristics, type of healthcare provider and patient related factors influencing adherence and social support. Adherence was measured using pill counting, as well as a self-reporting method adapted from a South African study [7]. Good adherence was defined as mothers who did not miss any antiretroviral (ARV) drugs in the last four weeks prior to the interview and who responded correctly to at least three of the four self-report questions. Poor adherence was defined using the pill-counting method, as missing more than one pill within a month (4 weeks). Knowledge of respondents was graded using twenty-five questions, for each correct response; one point was scored, and zero for wrong response. The average knowledge score was determined. Persons scoring the average score, or more were classified as having good knowledge while those scoring below the average score were termed to have poor knowledge. Data was collected using a pre-tested interviewer administered questionnaire. Seven research assistants were recruited and trained on how to obtain informed consent from participants and how to improve response rate. They were trained also on how to effectively administer the questionnaire in order to obtain quality data. Data was edited, cleaned, coded, entered and analyzed using Epi-Info version 7.2.0.1 and Microsoft Excel. Descriptive statistics were calculated to determine the level of adherence and factors influencing adherence. Bivariate analyses were done to determine the presence of statistically significant associations between independent variables and adherence level. Multiple logistic regression was conducted to control for confounding and identify factors that were independently associated with adherence to Option B+ antiretroviral therapy. All the statistically significant independent variables from the bivariate analyses were included in the logistic model. The logistic model coefficients were determined to be significant if their p value was ≤ 0.05 on the Wald Chi squared test. Ethical approval was received from the Federal Capital Territory Health Research Ethic Committee prior to conducting the study. The purpose of the study was explained to each participant and a written informed consent was signed by each participant before data were collected. Participation was voluntary and confidentiality was maintained.

## Results

A total of 284 HIV positive pregnant women and lactating mothers receiving PMTCT services participated in the study. One hundred and forty-seven (51.7%) were lactating mothers while 137 (48.3%) were pregnant. The mean age of respondents was 30.1 years (SD±4.9), with majority (71.5%) of the respondents within the age group 25-34 years. Of all the respondents, 272 (95.8%) were married. Twenty (7.0%) respondents had no formal education, 140 (49.3%) had secondary education, while 86 (30.28%) had tertiary level of education (Table 1). The level of adherence by pill count was adjudged to be 82.3%. Two hundred and thirty-three (82.3%) of the respondents had ≥ 95% adherence to Option B+ ART, 47 (16.5%) had difficulties in remembering to take their medication, while 20 (7.04%) usually stop taking their drugs when they feel better. Eleven respondents (3.87%) also reported that they stop taking drugs when they feel worse from side effects and 31(10.92%) missed at least one dose of ARVs in the last 3 days preceding the interview (Table 2).

**Table 1:** socio-demographic characteristics of women who uptook option B+ in healthcare facilities in Abuja, 2017

Variables	Frequency (n=284)	Proportions (%)
**Age Group (in years)**		
**15-25**	29	10.2
**25-34**	203	71.5
**35-44**	48	16.9
**≥ 45**	4	1.4
**Marital status**		
**Single**	7	2.5
**Married**	272	95.8
**Divorced**	2	0.7
**Widowed**	3	1.1
**Religion**		
**Christianity**	232	17.9
**Islam**	51	0.4
**Traditional**	1	30.3
**Level of education**		
**Higher/Tertiary**	86	30.3
**No formal education**	20	7.0
**Primary**	26	402
**Secondary**	140	9.2
**Technical/Vocational**	12	4.2
**Occupation**		
**Government employee**	27	9.5
**House wife**	91	32.0
**Business woman**	59	20.8
**Artisans**	31	10.9
**Private employee**	75	26.4
**Student**	1	0.4
**Place of residence**		
**Rural**	152	53.5
**Urban**	132	46.5
**Respondent status**		
**Lactating mother**	147	51.8
**Pregnant**	137	48.2

**Table 2:** level of adherence of respondents to option B+ ART in healthcare facilities in Abuja, 2017

Variables	Frequency(n=284)	Proportions(%)
**Level of adherence**		
Good adherence (≥95)	233	82.3
Poor Adherence (<95)	50	17.7
**Difficulty in remembering to take medication**		
No	237	83.4
Yes	47	16.6
**Stopping medication when better**		
No	264	93.0
Yes	20	7.0
**Missed a dose of ARV in the last 3 days**		
No	264	93.0
Yes	20	7.0
**Stopping medication when feeling worse**		
No	273	96.1
Yes	11	3.9

Bivariate analysis showed there is a significant association between educational level and adherence. Patients with formal education were three times (OR: 3.18; C.I: 1.36-7.44) more likely to be adherent to Option B+ ART compared with respondents with no formal education. There was however, no statistically significant association found between age of respondents <30 years (OR: 0.88; C.I: 0.43-1.82), place of residence (OR: 1.08; C.I: 0.58-1.97), marital status (OR: 1.07; C.I: 0.23-5.07), woman´s current status - pregnant or lactating (OR: 1.08; C.I: 0.59-1.99), and adherence. This study also showed a statistically significant association between patient-related factors such as disclosure of status (OR: 3.08; C.I: 1.54-6.14), Having good knowledge (OR: 2.12; C.I: 1.12-4.01), feeling depressed (OR: 17.80; C.I: 6.52-48.64), time taken to reach healthcare facility (OR: 2.88; C.I: 1.32-6.28), being too busy (OR: 23.38; C.I: 9.14-59.77), religious functions such as fasting (OR: 14.81; C.I: 1.51-145.48), lacking food to eat (OR: 20.17; C.I: 2.43-184.65), experiencing side effects (OR: 18.80; C.I: 3.78-93.58), forgetfulness (OR: 17.92; C.I: 8.53-37.65) and adherence. No statistically significant relationship was found between adherence and patients-related factors such as CD4 count at initiation of management (OR: 1.49; C.I: 0.67-3.31) and level of partner support (OR: 1.21; C.I: 0.65-2.25) (Table 3, Table 4).

**Table 3:** association between respondent’s socio-demographic factors and adherence to option B+ ART in healthcare facilities in Abuja, 2017

Variable	Level of Adherence		OR (Confidence
	Good Adherence (n=233)	Poor Adherence (n=50)	Interval)
**Age of Respondents**			
<30	109(83.4%)	21(16.6%)	0.9 (0.4-1.8)
≥30	124(81%)	29(19%)	
**Place of residence**			
Rural	125(82.8%)	26(17.2%)	
Urban	108(81.8%)	24(18.2%)	1.08 (0.6-1.9)
**Marital status**			
Not Married	10(83.3%)	2(16.7%)	
Married	223(82.3%)	48(17.7%)	1.07 (0.2-5.1)
**Educational level**			
Formal education	216(84.4%)	40(15.6%)	
No formal education	17(63%)	10(37%)	3.18 (1.4-7.4)*
**Woman’s Status**			
Pregnant	112(81.8%)	25(18.2%)	
Lactating	121(82.9%)	25(17.1%)	1.08 (0.6-2.0)
**Time taken to reach Healthcare Facility**			
≤ 1 hour	210(84.7%)	38(15.3%)	
> 1 hour	23(65.7%)	12(34.3%)	2.9 (1.3-6.3)*

* Statistically significant factors

**Table 4:** patient-related factors associated with adherence to option B+ ART in healthcare facilities in Abuja, 2017

Variable	Level of adherence		Odds Ratio
	Good adherence (n=233)	Poor adherence (n=50)	
**Disclosure of status**			
Yes	197(85.7%)	33(14.3%)	
No	33(66.0%)	17(34.0%)	3.08(1.5-6.1)*
**Knowledge score**			
Good knowledge	137(86.7%)	21(13.3%)	
Poor knowledge	80(75.5)	26(24.5%)	2.12(1.1-4.0)*
**Feel depressed**			
No	227(87%)	34(13%)	
Yes	6(27.3%)	16(72.7%)	17.86.5-48.6)*
**Too busy**			
No	226(88.6%)	29(11.4%)	
Yes	7(25%)	21(75%)	23.4(9.1-59.8)*
**Fastin**			
No	232(83.2%)	47(16.8%)	
Yes	1(25%)	3(75%)	14.8(1.5-145.5)*
**Level of partner support**	140(82.4%)	29(17.6%)	
Good Support	140(82.4%)	29(17.6%)	
Poor Support	77(81.1%)	18(18.9%)	1.2(0.7-2.3)
**CD4 count at initiation**			
≥ 350	59(83.1%)	12(16.9)	
<350	66(76.6%)	20(23.4%)	
**Side effects**			
No	231(84.3%)	43(15.7%)	
Yes	2(22.2%)	7(77.8%)	18.8(0.7-3.3)
**Lack of food**			
No	232(83.5%)		
Yes	1(20%)	4(80%)	20.2(2.4-184.7)*
**Pill burden**			
No	233	46	
Yes	0(0%)	4(100%)	Undefined
**Forgetfulness**			
No	215(91.5%)	20(8.5%)	
Yes	18(37.5%)	30(62.5%)	17.9(8.5-37.7)*
**Clinic not accessible**			
No	233(82.9%)	48(17.1%)	
Yes	0(0%)	2(100%)	Undefined
**Lack of care/support**			
No	233(84.1%)	44(15.9%)	
Yes	0(0%)	6(100%)	Undefined

* Statistically significant factors

Bivariate analysis of the association between provider-related factors influencing adherence shows a statistically significant relationship adherence and healthcare providers informing client of possibility of side effects (OR: 4.52; C.I: 2.09-9.76) and encountering challenges during diagnosis or ART initiation (OR: 2.3; C.I: 1.02-5.16). No association was found between adherence and clients´ waiting time at ART clinic (OR: 0.57; C.I: 0.29-1.15), number of counselling sessions (OR: 0.98; C.I: 0.53-1.18) and relationship with healthcare providers (Table 5). Logistic regression was performed to control for any confounding. The model included all factors that were analyzed and statistically significant at bivariate analysis. Time taken to reach health facility (OR: 2.88; C.I: 1.12-8.16), having side effects (OR: 39.63; C.I: 4.46-352.32), Lacking food to eat (OR: 34.76; C.I: 2.37-509.33), forgetfulness (OR: 20.02; C.I: 6.42-62.48), disclosure of HIV status (OR: 2.51; C.I: 1.22-5.15), being too busy (OR: 0.98; C.I: 0.53-1.18), being depressed (OR: 15.82; C.I: 3.48-71.89), level of client knowledge (OR: 2.12; C.I: 1.06-5.42) and being told about possibility of developing side effects (OR: 4.02; C.I: 1.85-4.72) were found to be significant predictors of adherence in our study (Table 6).

**Table 5:** provider related factors that are associated with adherence to option B+ ART in healthcare facilities in Abuja, 2017

Variable	Level of adherence		Odds Ratio (Conf. Int)
	Good adherence (n=233)	Poor adherence (n=50)	
**Waiting time**			
<3 hours	134(79.8%)	34(20.2%)	
≥3 hours	89(86.4%)	14(13.6%)	0.6(0.3-1.2)
**Told of side effects**			
Yes	113(92.6%)	9(7.4%)	
No	111(73.5%)	40 (26.5%)	4.5(2.1-9.8) *
**Counselling session**			
≥ 3	97(82.2%)	21(17.8%)	
<3	132(82.5%)	28(17.5%)	1.0(0.5-1.2)
**CD4 count at initiation**			
≥ 350	59(83.1%)	12(16.9%)	
<350	66(76.7%)	20(23.3%)	1.5(0.7-3.3)
**Challenges in ART initiation**			
No	70(89.7%)	8(10.3%)	
Yes	156(79.2%)	41(20.8%)	2.30(1.02-5.2)*
**Relationship with healthcare providers**			
Good	169(82.4%)	36(17.6%)	
Poor	47(82.5%)	10(17.5%)	0.99(0.5-2.2)

* Statistically significant factors

**Table 6:** predictors of adherence to option B+ ART in healthcare facilities in Abuja, 2017

Variable	Adjusted OR	95% CI
**Educational level**	**1.2**	**0.3-4.2**
Time taken to reach health facility	2.9	1.1-8.2*
Having side effects	39.6	4.5-352.3*
Lack of food	34.8	2.4-509.3*
Forgetfulness	20.0	6.4-62.5*
Disclosure of HIV status	2.5	1.2-5.2*
Being too busy	13.9	3.9-49.9*
Being depressed	15.8	3.5-71.9*
**Fasting**	**6.8**	**0.6-79.5**
Level of knowledge	2.1	1.1-5.4*
Counselled on side effects	4.0	1.9-4.7*
Challenge in diagnosis and ART initiation	2.1	1.0-4.7

* Predictors of adherence

## Discussion

We found the level of adherence to option B+ ART to be 82.3% among the respondents of our study in Abuja, Nigeria. Our finding is higher than the findings in Nnewi (78.3%) [8], Lagos in Nigeria (80.6%) [9] but lower than the findings of Ebuy et al in Tigray region, Northern Ethiopia (87.1%) [6] and Yirgalem in South Ethiopia. It is also slightly lower than that reported from a study conducted in Bwaila Hospital, Malawi (91%). The differences in results may be attributable to the different methodology used for assessing the level of adherence. In the Malawi study, the researchers used the pill count from the electronic medical record system; this was not done in our study and may account for the difference in adherence level. This study shows that majority of the participants were in the age group 25 to 34 years (71.5%), followed by 16.9% in the age group 35 to 44 years. This was similar to the study by Ebuy *et al*. [6] but differs from the study by Pennap *et al*. [10] in Keffi, Nassarawa State. The mean age of respondents was 30.1 years (SD±4.9). The mean age of the respondents was lower to that observed in Sokoto by Agu *et al*. Uzochukwu et al in Enugu and Wasti *et al*. [10] in Nepal. Furthermore, our observation was lower than what has been reported by Olowookere *et al*. [11] in Ibadan, Potchoo *et al*. in Togo [12] and Golin *et al*. [10]. This might be due to the fact that the other studies were carried out in the general population. Of the respondents, 272 (95.8%) were married. Twenty (7.0%) had no formal education while majority 140 (49.3%) had secondary education and 86 (30.3%) had tertiary level of education. One hundred and forty-seven (51.8%) were lactating mothers while 137 (48.3%) were pregnant.

No significant differences in the adherence levels of mothers who lived in rural areas to option B+ PMTCT drugs when compared to those who lived in urban areas. This is in contrast to studies carried out in Arba Minch General Hospital, southern Ethiopia and Dubti Hospital, east Ethiopia. This may be due to differences in socio-demographic characteristics of the study population and also that our study area has many sub-urban health facilities that rural dwellers can easily access. HIV-positive pregnant women having no formal education, those who took more than one hour to reach health facility, experienced side effects to antiretroviral drugs, Lacked food to eat, those who forgot to take their drugs, those who were too busy, those who had not disclosed to their partner or family, those who felt depressed, those with poor knowledge of HIV with PMTCT, those who were not counselled on the possibility of developing side effects to ARVs and breastfeeding mothers who faced challenges in testing and initiating option B+ treatment on the same-day as their HIV diagnosis tended to exhibit poor adherence. Not many studies have shown these relationships, but a study carried out among Malawian women described that feeling pressure to initiate ART immediately at the time of HIV diagnosis with little or no support in health-related decision-making was significantly associated with poor adherence. As with any interview study, the data was subject to recall bias. Although an effort was made to limit recall of medication to 30 days before the interview, it was possible that subjects over- or underestimated their adherence to ART. Non-use of virological and immunological laboratory investigation to corroborate self-reports in assessing adherence. Finally, the time for collection of data was only 10 weeks, which may limit generalization of our result since other patients could have different disclosure patterns although we have no reason to believe this is the case.

## Conclusion

The level of adherence to ART was high, but has not reached the optimum desirable level of 95%. The patient-related factors that were associated with adherence to option B+ ART include: fear of stigma/ disclosure of HIV status, being too busy, feeling depressed, lack of food and forgetfulness. Other patient-related factors include level of education, level of knowledge and the time it takes the patient to reach the health facility. Provider related factors associated with adherence include being counseled about possible side effects of antiretroviral drugs and facing challenges during diagnosis or initiation of antiretroviral therapy. Based on our study, we recommended that Public health department of the Federal Capital Territory in collaboration with development partners like IHVN and AFENET to organize public enlightenment on HIV/AIDS through the media which may help reduce stigma and encourage voluntary HIV status disclosure thereby improving adherence. Health care provider should provide in-depth adherence counseling and health workers who work in PMTCT clinic should allow adequate time for decision making to initiate option B+ lifelong treatment because option B+ drugs is not an emergency treatment. The National AIDS and STI Control Program (NASCP) and National Agency for the Control of AIDS should conduct more research studies on adherence from time to time to measure adherence level of patients on Option B+ antiretroviral. Health worker should counsel about use of different techniques like use of reminder, male partner or family support to minimize forgetfulness. They should also counsel patients on the need to seek help for depression and help refer them to the right place. The Federal Capital Development Agency (FCDA) Abuja and Federal Government of Nigeria should create job opportunities and skills acquisition through poverty alleviation programs in order to increase income of the people thereby alleviating poverty.

### What is known about this topic

- Option B+ antiretroviral therapy can reduce risk of mother-to-child transmission of HIV to 1-2% as against 15-40% without treatment what of the other treatment option? Quote the reduction of risk it can contribute to;
- Poor adherence is mostly responsible for poor treatment outcomes among people receiving antiretroviral therapy;
- Most new infections of HIV occur in Africa with mother to child transmission accounting for 90% of such infections Reorder.

### What this study adds

- The level of adherence to Option B+ ART among HIV positive pregnant women and lactating mothers in Abuja is 83.3%;
- Fear of stigma/disclosure of HIV status, being too busy, feeling depressed, lack of food and forgetfulness are patient-related factors influencing adherence to ART;
- Counseling clients before commencement of ART positively influence adherence.
